# Monitoring Respiratory Health in Children With Acute Asthma Using Wearable Electrical Bioimpedance and Breath Sounds: Observational Case-Control Study

**DOI:** 10.2196/72979

**Published:** 2026-03-05

**Authors:** Jesus Antonio Sanchez-Perez, John Berkebile, Natalie Jordan, Kevin Maher, Omer Inan, Jocelyn Grunwell

**Affiliations:** 1 Department of Electrical and Computer Engineering College of Engineering University of Puerto Rico-Mayaguez Mayagüez Puerto Rico; 2 School of Electrical and Computer Engineering College of Engineering Georgia Institute of Technology Atlanta, GA United States; 3 Division of Critical Care Medicine Children's Healthcare of Atlanta Atlanta, GA United States; 4 Department of Pediatrics School of Medicine Emory University Atlanta, GA United States

**Keywords:** asthma, impedance pneumography, electrical bioimpedance, lung sounds, wearable, multimodal sensing

## Abstract

**Background:**

Asthma remains one of the most serious chronic diseases of childhood. Individuals with severe asthma experience sudden episodes of breathlessness due to acute airflow obstruction, leading to recurrent pediatric intensive care unit (PICU) admissions that often result in mechanical ventilation and even death. Existing clinical assessments lack temporal resolution to effectively track the rapidly changing physiology.

**Objective:**

This study aimed to evaluate the feasibility of quantifying respiratory health during acute asthma in children using wearable multimodal sensing.

**Methods:**

Wearable-based impedance pneumography (IP) and multichannel lung sounds (LSs) were measured on 17 children admitted to the PICU with an acute asthma attack and on 9 healthy controls. Short-term multimodal measurements were obtained throughout hospitalization, specifically at PICU admission (T1) and discharge (T2). Measurements were also obtained from controls without any signs of acute asthma or otherwise healthy. Statistical and clustering analyses were performed to identify trends in IP- and LS-derived respiratory markers from T1 to T2 across all patients with paired time points (n=13), as well as across the matched cohort (T1: n=10 and T2: n=7), who were compared against controls (n=9). Five features were computed from the IP signal: respiratory rate, inspiration time (Ti), expiration time (Te), expiration-to-inspiration time ratio (Te:Ti), and the normalized Ti by interbreath interval (Ti/IBI). Leveraging the breathing context provided by the IP signal, 4 spectral integrated intensity (SI) acoustic features were computed in 4 different subbands for the inspiration and expiration phases.

**Results:**

Within the patient group (n=13), we found that respiratory rate decreased (*W*(12)=79; *P*=.02), whereas Te (*W*(12)=12; *P*=.02) and Ti (*W*(12)=13; *P*=.02) lengthened. Meanwhile, the SIs for the lowest subband (100-300 Hz) decreased for both inspiration and expiration phases (*P*<.01), while they increased for the highest subband (800-1000 Hz) for both inspiration and expiration phases (*P*<.01). Significant differences also existed between T1 and control and T2 and control of the matched cohort. We found that all features were significantly different between T1 and control (*P*<.05), and all SIs together with Te:Ti and Ti/IBI were significantly different between T2 and control (*P*<.05), all exhibiting trends toward normalcy.

**Conclusions:**

These results demonstrate the feasibility of quantifying and tracking respiratory health in children with acute asthma using wearable multimodal sensing, specifically with the fusion of IP- and LS-derived markers. Such technology may provide a new adjunctive clinical tool for real-time respiratory monitoring and enable timely titration of care, thereby improving patient outcomes.

## Introduction

Asthma affects more than 4.6 million (6.5%) of US children, resulting in more than 270,000 emergency department visits and 27,000 hospitalizations for status asthmaticus annually [1,2]. Individuals with asthma experience recurrent episodes of reversible airflow obstruction, resulting in breathlessness, chest tightness, wheezing, and coughing. Severe asthma is characterized by the sudden worsening of these symptoms (asthma attacks) and trapping of air inside the lungs (air trapping) due to bronchoconstriction [3]. While many children respond to emergency treatment with bronchodilators and systemic corticosteroids, others progress to respiratory failure and require admission to a pediatric intensive care unit (PICU). PICU admissions for respiratory failure have increased in proportion to general hospital admissions for asthma [4] for reasons that are unclear. Children with severe asthma can have recurrent PICU admissions due to exacerbations that may require mechanical ventilation and even result in death.

Management of asthma involves, among other strategies, the use of inhaled corticosteroids and β-agonist therapy to reduce airway inflammation and promote bronchodilation, thereby reversing airflow obstruction and improving symptoms [5]. Titration of care commonly relies on symptom reporting, physical examination, and pulmonary function tests [3,5]. However, these assessments are often highly subjective and lack the temporal resolution needed to capture the rapidly evolving physiology of a disease characterized by substantial interindividual variability in treatment response [6,7]. On physical examination, the hallmark of asthma monitoring is the manual evaluation of lung sounds (LSs) via auscultation with a stethoscope [3,8]. Unfortunately, this technique is limited by its manual, qualitative, and subjective nature [8]. Digital auscultation enables the quantitative acoustic analysis of LSs, but commercially available electronic stethoscopes do not provide breathing-phase context for post hoc analysis and only record for a short amount of time at a single location. Pulmonary function test via spirometry, on the other hand, requires patient cooperation for performing respiratory maneuvers, which may not be feasible in young children [9], and these diagnostic maneuvers cannot be performed during an acute exacerbation. Hence, there is a need for noninvasive sensing technologies that can consistently quantify asthma signs continuously and conveniently.

Wearable noninvasive sensing systems have emerged as promising technological approaches for the continuous monitoring of the physiological manifestations of asthma [10,11]. These include vital sign monitoring via electrocardiogram, photoplethysmogram [12], LSs [13], chest movement [14], and electrical bioimpedance (BioZ) signals [15,16]. Yet, demonstrations of these technologies in pediatric populations are scarce. Specifically for children with asthma, van der Kamp et al [17] proposed a suite of sensors for at-home monitoring that leveraged an electrocardiogram for heart rate and respiratory rate (RR) estimation. An at-home sensing suite was also proposed by Hosseini et al [18], including a portable spirometer and heart rate derived from a smartwatch. Despite the promising results, these technologies only capture a limited number of markers that may not fully capture the complex physiological manifestations of asthma.

Recent studies have proposed the use of impedance pneumography (IP) as a promising noninvasive sensing modality for respiratory flow and volume signal measurement in children with asthma [19-21]. Prior work has demonstrated the accurate estimation of respiratory markers such as RR, breathing-phase timings, tidal volume and flow, among others, in both laboratory and clinical settings [15,19,20,22-25]. Notably, we recently demonstrated the feasibility of measuring IP simultaneously with multichannel respiratory sounds in a clinical setting and studied their joint application for continuous breathing-phase contextualization in computerized respiratory sound analysis [26,27]. Given the critical role of auscultation and lung function assessment in asthma management, the joint application of such technologies for the continuous monitoring of severe asthma signs in children warrants further investigation.

**Figure 1:**
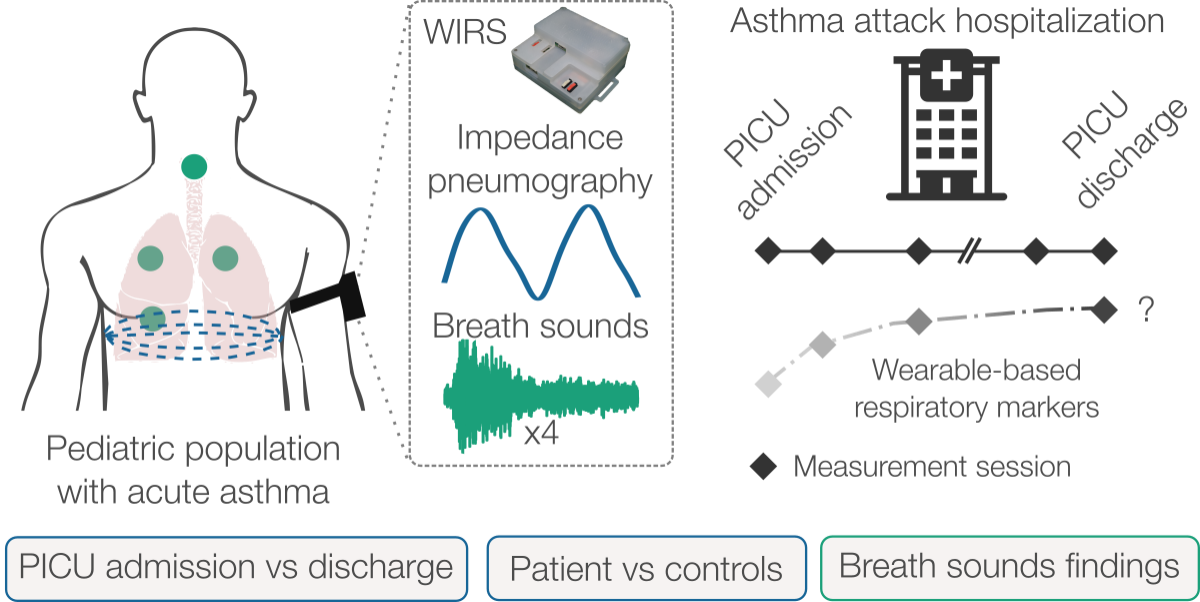
Overview of the study. Transthoracic electrical bioimpedance and multichannel breath sounds were simultaneously measured on children admitted to the PICU with an asthma attack using our WIRS. Measurements were taken after PICU admission and before discharge. The feasibility of using these signals for monitoring asthma symptoms was evaluated by comparing admission-to-discharge feature trends, differences between patients and controls, and breath sound findings. PICU: pediatric intensive care unit; WIRS: wearable impedance pneumography and respiratory sound system.

This study aimed (1) to evaluate the feasibility of using respiratory markers derived from wearable-based transthoracic BioZ and LSs to quantify the physiological manifestations of life-threatening asthma from peak to resolution of signs and (2) to compare their trajectory against healthy controls, as illustrated in Figure 1. To this end, we evaluated multimodal measurements from 17 children admitted to the PICU with an acute asthma attack, as well as 9 healthy controls. We took measurements up to twice daily during hospitalization and carried out several statistical analyses to assess trends in the wearable-based multimodal respiratory markers from PICU admission to discharge and compared them against controls. Ultimately, the combination of BioZ-derived respiratory markers and acoustic signatures derived from the LSs in a wearable form factor may provide clinicians with a new adjunctive clinical tool for real-time respiratory monitoring and enable timely titration of care, thereby improving patient outcomes.

## Methods

### Human Subjects Study

Eligible participants for the patient group were participants admitted to the PICU at the Children’s Healthcare of Atlanta (CHOA), Atlanta, Georgia, with a severe asthma attack. Exclusion criteria included substantial comorbidity (eg, cystic fibrosis, bronchiectasis, and pulmonary aspiration). Children were enrolled irrespective of a prior diagnosis of asthma because there are some children whose first presentation of symptoms requires hospitalization, sometimes in the PICU [28]. Eligible participants for the control group were participants with no prior history of respiratory illness as the cause of intensive care admission and without wheezing or otherwise healthy, nonhospitalized individuals. All participants were in the age range of 1-17 years. Measurements took place between April 2023 and April 2024.

### Ethical Considerations

The human subject study was carried out with the approval from the Emory University School Institutional Review Board (STUDY0004316) and the Georgia Institute of Technology (IRB #H22329). Informed consent was obtained from the participants’ parents or legal guardians prior to any study procedures by trained pediatric research coordinators. Additionally, per institutional review board requirements, children aged 6-11 years provided oral assent and those aged 12-17 years provided written assent, when feasible, given their medical condition. All metadata and physiological signals collected at the CHOA site were properly deidentified before sharing for further processing. No compensation was provided to the participants of this study.

### Experimental Protocol

Following enrollment, noninvasive physiological measurements were obtained in the morning (AM) or afternoon (PM), whenever possible, while the participants remained hospitalized in the PICU. The first and last measurements in the PICU are hereafter referred to as T1 and T2, respectively. For healthy participants in the control group, a single measurement took place in a hospital staff room within the PICU, with the participants in the sitting position. For participants in the control group who were hospitalized without signs and symptoms of acute asthma, 2 measurements were taken, and only the first one was used for further analysis. Recordings lasted up to 15 minutes, determined by the cooperativeness of the participants, following which all sensors were detached from the participant, and the recordings were saved for further analysis. No instructions were provided to any of the participants to modulate or control their breathing patterns for the recordings. Clinical data from the electronic medical record were also collected in each recording.

### Physiological Measurement

Our custom wearable impedance pneumography and respiratory sound system (WIRS) was used in this study to simultaneously measure transthoracic BioZ and LSs [26]. Transthoracic BioZ was measured at 4 excitation frequencies (5, 50, 100, and 150 kHz). Each frequency was sampled at 16 Hz and calibrated to form complex impedance vectors [29]. A tetrapolar electrode configuration was used, with a pair of current-injecting electrodes placed along the mid-axillary line approximately at the fifth intercostal space and the voltage-measuring electrodes in the same vertical plane and separated by 5 cm approximately. Standard 3M Ag/AgCl gel electrodes were used. This BioZ sensing module was previously validated in prior work [22,23,26].

LSs were measured at 4 locations simultaneously using WIRS. Three locations were used to measure LSs: the anterior right and left upper chest and the posterior right lower chest. The fourth channel was moved from the posterior left lower chest to the suprasternal notch or neck for some participants to measure tracheal sounds. As in prior work, the accelerometer contact microphones (BU-23173-000, Knowles Electronics LLC) were enclosed in our previously reported acoustic enclosure and secured with surgical tape [26]. For the youngest participants, due to their small thorax sizes and for tracheal sound measurements, a more compact acoustic enclosure was used. These consisted of the same overmolded microphones used in Sanchez-Perez et al [26], but only a 3D-printed hollow backing piece was used to prevent loading of the sensor when external forces are exerted on it. Lying over the sensors for posterior chest auscultation while in the supine position is a notable example of such a case. The effectiveness of this acoustic solution was validated in prior work [30].

To validate the recordings against a commercial stethoscope, simultaneous recordings were taken with the Eko CORE digital stethoscope (Eko Devices). The recordings were manually aligned with WIRS following the simultaneous tapping of the Eko CORE diaphragm and the proximal WIRS channel. All measurements were performed by clinical staff at CHOA.

### Signal Processing

#### Impedance Pneumography

**Figure 2:**
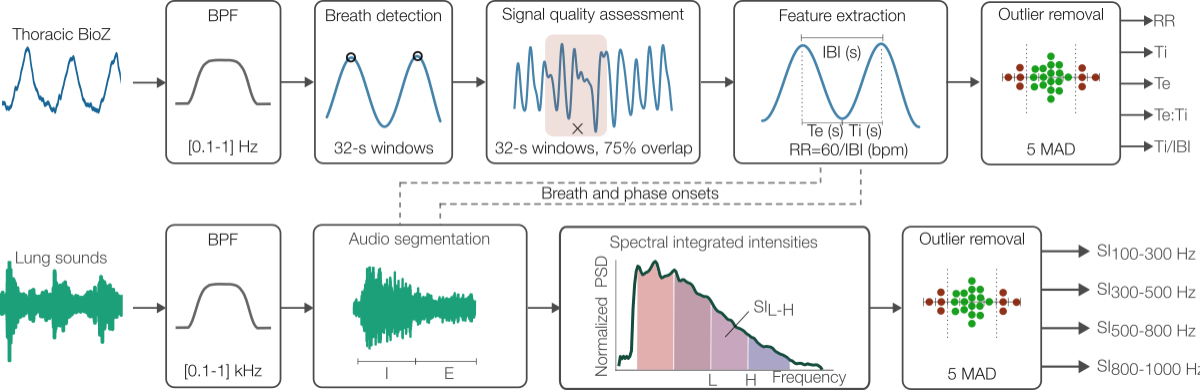
Multimodal signal processing pipeline used to compute breath-by-breath and phase-by-phase respiratory features. BioZ: electrical bioimpedance; BPF: bandpass filter; E: expiration; I: inspiration; IBI: interbreath interval; MAD: median absolute deviation; RR: respiratory rate; SI: spectral integrated intensity; Te: expiration time; Te:Ti: expiration-to-inspiration time ratio; Ti: inspiration time.

The IP signal processing pipeline used in this work is shown in Figure 2. IP is a noninvasive signal that measures the respiratory-mediated changes in thoracic BioZ and has demonstrated excellent correlation to spirometric airflow and volume measures [31,32]. The pipeline follows a similar structure to our previously published version [26] but was modified for this dataset, which took place in an acute-care setting with a pediatric population in the awake state. BioZ-based respiratory monitoring in children with asthma has been previously performed during sleep due to their notorious lack of cooperation, which represents a challenge for physiological measurements [19,20].

The multifrequency thoracic BioZ signals were first linearly resampled to 100 Hz using a finite impulse response (FIR) antialiasing low-pass filter [26]. An amplitude-based removal stage was then applied to the signals to remove transient events with abnormally high thoracic BioZ values. These events were observed in some participants, likely due to abrupt movements loosening the BioZ wire connection. In this stage, portions of the signal were flagged for removal if they were 1.5 above or 0.5 below the median BioZ. Finally, the removed values were filled via linear interpolation.

As shown in Figure 2, the BioZ signals (150 kHz excitation frequency) were bandpass filtered with a Kaiser window FIR filter in a nominal frequency range of 0.1-1 Hz (6-60 bpm), resulting in IP 150 kHz. The upper end was increased up to 1.4 Hz for some participants with clear dominant frequencies above 1 Hz to ensure accurate processing of the signals. Following filtering, interbreath intervals (IBIs), hereafter referred to as “breaths,” were detected on 32-second windows (2-second overlap) with a minimum distance between peaks set to 0.67 seconds (RR<89.55 bpm), using prior work methodology [33,34]. An RR of 90 bpm has been used as an extreme upper end in previous work [35]. A signal quality index was then assessed using the Charlton et al [36] method. The method was applied using 32-second windows with 75% overlap [15], wherein a breath was considered good if it was labeled as such in any of the windows it belonged to [26]. The normalized SD threshold (ie, coefficient of variation) of the signal quality index method was set to 0.3 to avoid excessive rejection with the original threshold of 0.25, as reported by Blanco-Almazan et al [15], while controlling for the high intraparticipant variability expected in the recordings. Further, a window was considered of good quality if the mean correlation to the window breath template was above 0.7 and at least 60% of the window contained valid breaths.

After signal quality assessment, 3 features were extracted on a breath-by-breath basis: RR, expiration time (Te), and inspiration time (Ti). A feature-based outlier removal stage was then performed to remove breaths if any of these features laid 5 median absolute deviation (MAD) away from the median, over a window of 30 breaths [26,34]. Following this stage, the final set of features was RR, Te, Ti, expiration-to-inspiration time ratio (Te:Ti), and the inspiration time normalized by the IBI duration (Ti/IBI). These respiratory markers have been widely studied for monitoring obstructive pulmonary diseases using BioZ [15,19,20] and spirometry [37].

#### Lung Sounds

The multichannel breath sounds were linearly resampled from 46.875 to 4 kHz to match Eko CORE sampling frequency using a FIR antialiasing low-pass filter. All LS signals were then filtered in the passband 100 to 1000 Hz using a zero-phase minimum-order FIR bandpass filter [27]. The low-frequency cutoff was chosen to suppress heart sound interference [38]. The upper cutoff frequency, on the other hand, suppressed high-frequency noise (eg, speech and hospital room noises) while preserving the parts of the spectrum occupied by normal and adventitious sounds (eg, wheezes) [39,40]. As in prior work, the IBI and phase (ie, inspiration and expiration) onset timings derived from the IP signal were used to index and segment the breath sounds [27]. Figure 2 shows an example of the process of fusing IP-derived timings with LS to segment the phases and extract features on them from prior work.

Following phase and breath segmentation, the normalized spectral integrated intensity (SI) described in Nabi et al [39] was computed across 4 different bands: 100-300, 300-500, 500-800, and 800-1000 Hz. These acoustic features capture the SI at frequency bands that have been found by prior airway obstruction modeling work [41] to be informative within the known LS frequency spectrum [40,42]. First, power spectral density (PSD) estimates were obtained using a 64-ms Hamming window [39] with 75% overlap. These were then normalized to 1 using their respective maximum PSD value to obtain PSD_norm_ [39]. The normalized SIs were then computed as the integrated PSD_norm_ in the specified ranges divided by total cumulative spectral intensity from 100 to 1000 Hz, as described in Nabi et al [39]. This was necessary to obtain comparable intensities regardless of the loudness of the sound, which depends on the airflow rate, auscultation location, and breathing phase [40,42]. The final SI values were defined as the average SIs across all windows within a given breathing-phase segment, resulting in SI100-300, SI300-500, SI500-800, SI800-1000, and for each range described earlier, respectively.

To assess outliers, the cumulative sum in the frequency range 100-1000 Hz of the unnormalized PSD of each breath segment and phase was used, namely, SI, SI_I_, and SI_E._ Specifically, a feature-based outlier removal stage was applied to remove breaths if any of these 3 features laid 4 MAD away from the median, over a window of 30 breaths. The final set of breaths used in this study consisted of the simultaneous good breaths following the processing procedures for IP and those described in this section for the LS data. In this way, the final breath instances had both IP and audio features. Hence, the final multimodal feature vector consisted of 5 IP features and 8 SI features across both breathing phases, yielding a total of 13 features.

#### Feature Time Series Generation

The final feature time series was generated after averaging the features over 32-second windows, 75% overlap to mitigate breath-by-breath variability due to physiology or phase-onset variability. A final outlier removal stage was performed by removing window averages lying 5 MAD from the median. Removed values were filled using linear interpolation. All signal processing was done in MATLAB R2020b (MathWorks).

### Statistical Analysis

Two separate statistical analyses were carried out to assess differences in respiratory timing features across time and between patient and control groups. In the former, differences in the features within participants with both T1 and T2 time points available were assessed using Wilcoxon signed rank paired statistical tests. In the latter, features from the control group were compared against T1 and T2 of the matched participants using Wilcoxon rank sum tests with Benjamini-Hochberg correction for 2 comparisons (ie, T2-control and T1-control). Comparisons against the control group were conducted to highlight a trend in improvement in respiratory features. Participants were retrospectively matched to controls based on BMI and weight. Before conducting statistical tests, the feature time series of each participant was first aggregated with their mean across any given time point. R5 kHz was excluded from the statistical analysis since the values may not be directly comparable between participants due to differences in electrode locations, posture, and thorax sizes. Differences in demographic data between matched participants and controls were also assessed using Wilcoxon rank sum tests. All statistical analyses were carried out in R (R Foundation for Statistical Computing). A 2-tailed *P* value <.05 was considered significant. Numerical data are shown as median and IQR, unless specified otherwise.

### Principal Component Analysis

The multidimensional feature matrix composed of IP- and LS-derived features was projected to 2 dimensions using principal component analysis (PCA) to elucidate any clustering between T1, T2, and control of the matched cohort. The first principal component (PC) was evaluated as a synthetic multimodal feature that maximally captures the variance in the data while retaining explainability. This analysis was carried out in Python (version 3.9; Python Software Foundation) using the scikit-learn library [43].

## Results

### Dataset Characteristics

Multimodal measurements were obtained from a total of 28 participants. The data from 2 participants were discarded due to measurement errors, resulting in 26 usable datasets (17 participants and 9 controls). Of the 9 controls, 7 were healthy participants, and 2 were patients hospitalized with no signs and symptoms of acute asthma. Table 1 shows detailed demographics for these participants, including the matched participants. No significant differences existed in the demographic data between matched participants and controls. Table 2 shows expanded demographic details for the participants, including the matched cohort, for T1 and T2. The variables include the type of respiratory support, albuterol administration (intermittent or continuous), terbutaline administration, aminophylline administration, time from the last albuterol administration, albuterol dosing interval, time to discharge from PICU, and time to discharge from hospital per recording (T1 and T2). A total of 48 multimodal recordings were obtained from 26 participants, from which 3 recordings were discarded due to poor data quality or measurement errors. The mean recording durations were 748 (SD 117), 661 (SD 86), and 688 (SD 111) seconds for T1, T2, and control, respectively. The mean proportion of raw BioZ data interpolated was 0.235% (SD 0.930%), 1.62% (SD 3.78%), and 0.146% (SD 0.327%) for T1, T2, and control, respectively. The mean final acceptable breaths retained for analysis were 76.1% (SD 23.4%) after applying our processing pipeline. The mean outlier removal proportion at the final feature time series level was 1.17% (SD 0.703%), 0.668% (1.06%), and 0.474% (SD 0.430%) for T1, T2, and control, respectively.

**Table 1:** Sample demographics.

			All patients (n=17)	Matched patients (n=10)		Controls (n=9)		*P* value^a^
**Sex, n (%)**								—^b^
	Male	11 (64.7)		6 (60)	4 (44.4)			
	Female	6 (35.3)		4 (40)	5 (55.6)			
Age (years), median (IQR)			9 (3-17)	6 (3-10)		5 (2-14)		.23
BMI (kg/m^2^), median (IQR)			18.6 (15.4-44.2)	17.1 (15.4-19.6)		17.0 (14.1-20.6)		.91
Height (cm), median (IQR)			137.0 (94-184)	118.5 (94-147)		101.0 (85-150)		.31
Weight (kg), median (IQR)			33.5 (16.4-123.6)	23.5 (16.4-33.9)		19.0 (12.1-46.3)		.18

^a^Wilcoxon rank sum test between matched patient and control demographic data.

^b^Not available.

**Table 2:** Patient demographics.

		All patients			Matched patients	
		T1 (n=17)	T2 (n=13)	T1 (n=10)		T2 (n=9)
**Type of support, n (%)**						
	No significant	8 (47)	12 (92)	7 (70)		6 (67)
	HFNC^a^	6 (35)	1 (8)	2 (20)		1 (11)
	BiPAP^b^	3 (18)	0 (0)	1 (10)		0 (0)
Intermittent albuterol, n (%)		15 (88)	9 (69)	8 (80)		3 (33)
Continuous albuterol, n (%)		2 (12)	2 (15)	2 (20)		2 (22)
Tertabuline, n (%)		4 (24)	0 (0)	2 (20)		0 (0)
Aminophylline, n (%)		1 (6)	0 (0)	0 (0)		0 (0)
Time from last albuterol (hours), median (IQR)		1.1 (0.4-7.5)	2.4 (0.2-18.6)	1.15 (0.4-7.5)		2.7 (0.2-18.6)
Albuterol dosing interval (hours), median (IQR)		2.5 (1.6-8.0)	4.3 (2.3-10.8)	3.0 (1.6-8.0)		3.9 (3.0-4.3)
Time to PICU^c^ discharge (hours), median (IQR)		22.9 (1.6-113.7)	4.3 (0.4-27.3)	9.5 (1.6-74.8)		5.0 (0.4-27.3)
Time to hospital discharge (hours), median (IQR)		46.1 (12.4-173.9)	27.3 (5.7-73.3)	28.0 (23.2-74.8)		26 (5.7-46.6)

^a^HFNC: high-flow nasal cannula (≥6 liters per minute).

^b^BiPAP: bilevel positive airway pressure.

^c^PICU: pediatric intensive care unit.

### Analysis of Multimodal Respiratory Features

**Figure 3:**
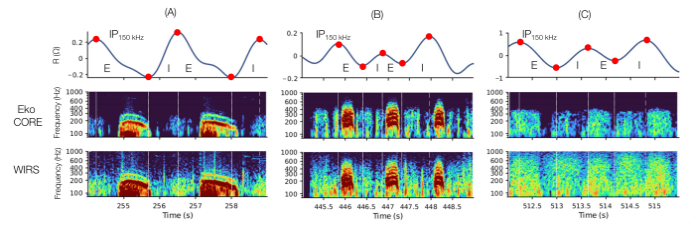
Exemplary simultaneous IP and lung sounds measurements. The top row shows the IP signal with annotated inspiration and expiration phase onsets. The spectrograms show proximal EKO CORE and WIRS recordings: (A) continuous abnormal expiratory sounds, (B) expiratory wheezes found in the afternoon session of a 3-year-old patient at T1, and (C) normal sounds measured the following morning (T2) on the same patient. The time from the last albuterol administration, albuterol dosing interval, time to hospital discharge, and time to PICU discharge were 1.75, 3.4, 26.6, and 26.6 hours, respectively, for (B) and 0.2,4.3, 5.7, and 5.7 hours, respectively, for (C). E: expiration; I: inspiration; IP: impedance pneumography; WIRS: wearable impedance pneumography and respiratory sound system.

Three exemplary multimodal recording segments are shown in Figure 3. The segments show the annotated IP signal along with spectrograms of LSs measured with the Eko CORE and the proximal WIRS channel. A transition from wheezing at T1 to normal LSs at T2 for a 3-year-old patient is shown in Figure 3B and 3C. The more compact acoustic enclosures described in the Physiological Measurement section were used due to the small thorax size. An exemplary full-length recording is illustrated in Multimedia Appendix 1. The recording shows the annotated IP signal providing respiratory context and a sharp transition in the phase-specific sound intensities computed for CH_3_ from strongly expiratory breath sounds shortly after the 150-second mark and back to normal-like strongly inspiratory sounds after 550 seconds. Exemplary spectrograms from all 4 channels during the segments with normal-like sounds and continuous strongly expiratory sounds are also illustrated.

**Figure 4:**
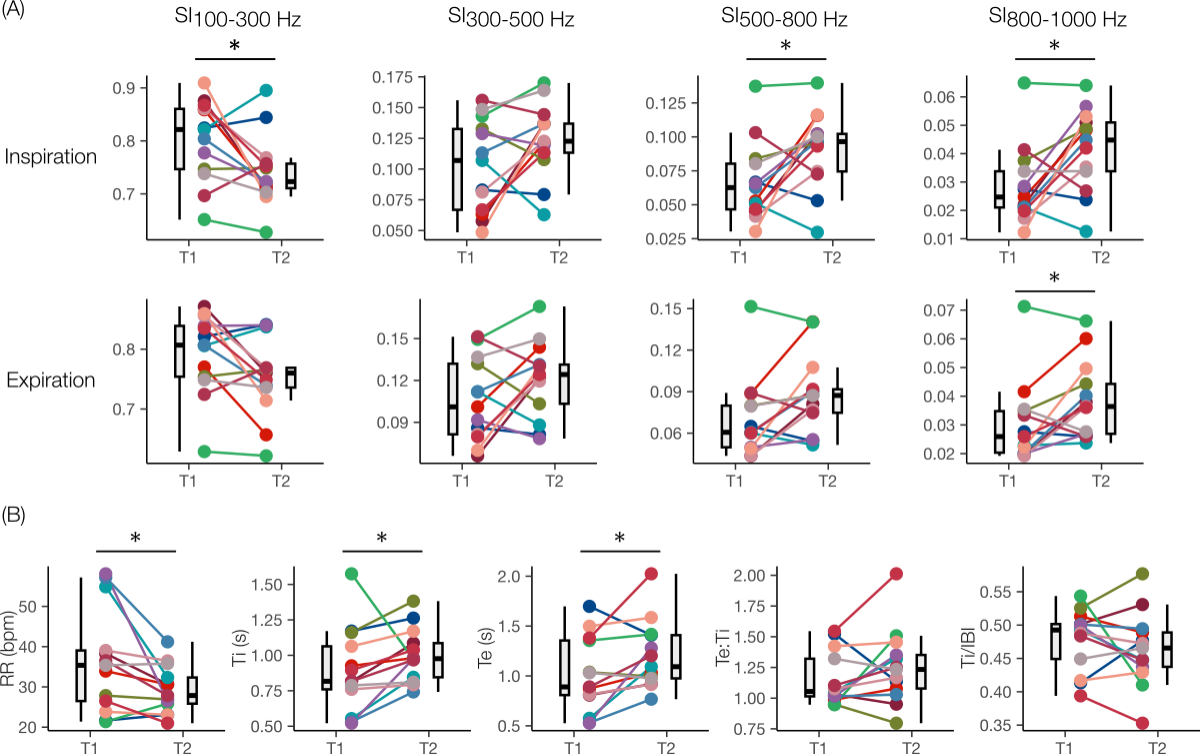
Boxplots of the changes in aggregated respiratory timing features from first (T1) to last (T2) measurement in the pediatric intensive care unit for all patients with paired time points (n=13). (A) SIs for the inspiration and expiration phase and (B) IP-derived respiratory timings. Patients are shown in different colors with lines connecting the mean aggregated data for the time point. IBI: interbreath interval; IP: impedance pneumography; RR: respiratory rate; SI: spectral integrated intensity; Te: expiration time; Te:Ti: expiration-to-inspiration time ratio; Ti: inspiration time. *P<.05.

The results for the paired time points of the participants are shown in Figure 4 and Table 3. Compared to T1, the IP-derived respiratory timings revealed a significant decrease in RR, while Ti and Te increased significantly. Te:Ti and Ti/IBI increased but did not reach statistical significance. The results for the LS-derived SIs revealed a decreasing trend for SI100-300 and an increasing trend for other subbands across both breathing phases. Significant differences were observed for SI100-300 in inspiration, SI500-800 in inspiration, and SI800-1000 in inspiration and expiration.

**Figure 5:**
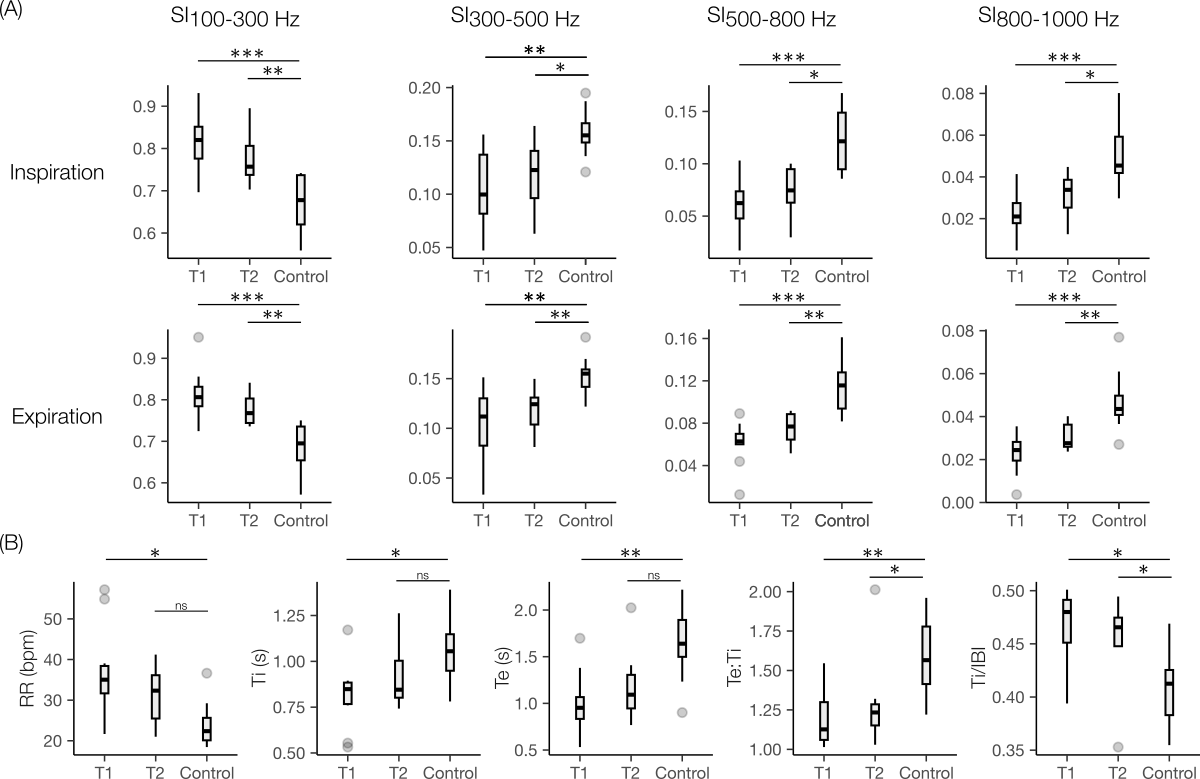
Comparison of multimodal features between first (T1, n=10) and last (T2, n=7) measurement in the pediatric intensive care unit and controls (n=9) for the matched cohort. (A) SIs for inspiration and expiration phase and (B) impedance pneumography–derived respiratory timings. IBI: interbreath interval; ns: not significant; RR: respiratory rate; SI: spectral integrated intensity; Te: expiration time; Te:Ti: expiration-to-inspiration time ratio; Ti: inspiration time. *P<.05, **P<.01, ***P<.001 after Benjamini-Hochberg correction.

The results for the comparison between patients and controls for the matched cohort are shown in Figure 5 and Table 4. Significant differences existed between T1 and control for all features. Further, all features except RR, Ti, and Te resulted in significant differences between T2 and control. For the respiratory timings, a decreasing trend toward the control distribution was observed in RR and Ti/IBI, while an increasing trend was observed for Ti, Te, and Te:Ti. For the LS features, a decreasing trend toward the control distribution was observed for SI100-300, while increasing trends were observed for the rest of the subbands. Further, for all groups (T1, T2, and control) and phases (inspiration and expiration), a decreasing trend from the first subband’s intensity (SI100-300) to the last (SI800-1000) was also observed (Table 4). Comparing T1 and control, the greatest change was observed for the inspiratory phase across all subbands, and the change magnitude increased as the frequency increased: SI100-300 (inspiration: –17.3% vs expiration: –13.77%), SI300-500 (inspiration: +55% vs expiration: +38.4%), SI500-800 (inspiration: +95.2% vs expiration: +84.1%), and SI800-1000 (inspiration: +114.3% vs expiration: +83.3%).

**Figure 6:**
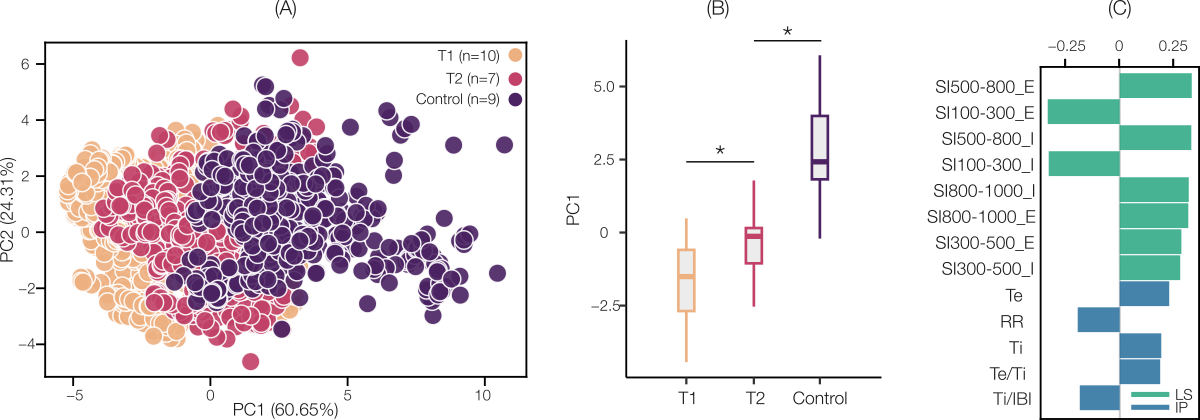
Principal component analysis results between the first (T1, n=10) and last (T2, n=7) measurement in the pediatric intensive care unit and controls (n=9) for the matched cohort. (A) Color-coded PC2 versus PC1 clustering, (B) boxplots for the comparison of PC1 values aggregated by patient, and (C) PC1 coefficients ranked in order of importance. E: expiration; I: inspiration; IBI: interbreath interval; IP: impedance pneumography; LS: lung sound; PC: principal component; RR: respiratory rate; SI: spectral integrated intensity; Te: expiration time; Te/Ti: expiration-to-inspiration time ratio; Ti: inspiration time. *P<.05 after Benjamini-Hochberg correction.

The PCA results for the matched cohort are shown in Figure 6. PC1 vs PC2 clustering and PC1’s loadings ranked in order of importance are shown in Figures 6A and 6C. The aggregated PC1 values per participant in the matched cohort (Figure 6B) resulted in significant differences between T1-control (*P*<.001) and T2-control (*P*=.003).

**Table 3:** Statistical summary of multimodal respiratory features for the patient group.

	T1^a^ (n=13), median (IQR)	T2^b^ (n=13), median (IQR)	*W* ^c^	*P* value^d^
SI100-300I^e,f^	0.821 (0.114)	0.723 (0.046)	75	.04
SI100-300E^g^	0.810 (0.085)	0.760 (0.033)	69	.11
SI300-500I	0.107 (0.066)	0.123 (0.024)	21	.09
SI300-500E	0.101 (0.051)	0.124 (0.028)	24	.15
SI500-800I	0.063 (0.034)	0.096 (0.028)	14	.03
SI500-800E	0.061 (0.030)	0.087 (0.017)	22	.11
SI800-1000I	0.025 (0.013)	0.045 (0.017)	15	.03
SI800-1000E	0.026 (0.036)	0.014 (0.017)	16	.04
RR^h^	35.37 (12.56)	27.86 (6.49)	79	.02
Ti^i^	0.817 (0.303)	0.976 (0.241)	13	.02
Te^j^	0.890 (0.548)	1.093 (0.433)	12	.02
TeTi	1.054 (0.308	1.234 (0.273)	26	.19
Ti/IBI^k^	0.493 (0.052)	0.465 (0.051)	57	.45

^a^T1: is the first measurement time point for the patients.

^b^T2 is the last measurement time point for the patients.

^c^*W* is the test statistic.

^d^Wilcoxon signed rank test between paired time points.

^e^SI: spectral integrated intensity.

^f^I: inspiration.

^g^E: expiration.

^h^RR: respiratory rate.

^i^Ti: inspiration time.

^j^Te: expiration time.

^k^IBI: interbreath interval.

**Table 4:** Statistical summary of multimodal respiratory features for the matched cohort.

	T1^a^ (n=10), median (IQR)	*W* ^b^	*P* value^c^	T2^d^ (n=9), median (IQR)	*W* ^b^	*P* value^c^	Control (n=7), median (IQR)
SI100-300I^e,f^	0.820 (0.075)	5	.001	0.757 (0.069)	6	.005	0.678 (0.117)
SI100-300E^g^	0.806 (0.047)	4	.001	0.768 (0.059)	5	.003	0.695 (0.081)
SI300-500I	0.100 (0.055)	81	.004	0.123 (0.044)	53	.02	0.155 (0.018)
SI300-500E	0.112 (0.048)	82	.003	0.124 (0.027)	57	.005	0.155 (0.017)
SI500-800I	0.062 (0.026)	87	<.001	0.075 (0.032)	55	.01	0.121 (0.054)
SI500-800E	0.063 (0.010)	89	<.001	0.077 (0.024)	60	.001	0.116 (0.034)
SI800-1000I	0.021 (0.010)	87	<.001	0.034 (0.013)	53	.02	0.045 (0.017)
SI800-1000E	0.024 (0.009)	86	.001	0.028 (0.010)	58	.003	0.044 (0.009)
RR^h^	35.038 (6.74)	13	.02	32.34 (10.65)	15	.09	22.37 (5.53)
Ti^i^	0.848 (0.12)	77	.02	0.85 (0.20)	45	.17	1.06 (0.20)
Te^j^	0.952 (0.23)	79	.01	1.09 (0.36)	49	.07	1.64 (0.40)
TeTi	1.126 (0.24)	80	.01	1.23 (0.13)	51	.04	1.57 (0.36)
Ti/IBI^k^	0.480 (0.04)	12	.01	0.47 (0.03)	12	.04	0.41 (0.04)

^a^T1: is the first measurement time point for the patients.

^b^*W* is the test statistic.

^c^Wilcoxon rank test between matched patient and control demographic data after Benjamini-Hochberg correction.

^b^T2 is the last measurement time point for the patients.

^e^SI: spectral integrated intensity.

^f^I: inspiration.

^g^E: expiration.

^h^RR: respiratory rate.

^i^Ti: inspiration time.

^j^Te: expiration time.

^k^IBI: interbreath interval.

## Discussion

### Principal Findings

In this study, we evaluated the feasibility of quantifying the physiological manifestations of acute asthma in children using a wearable multimodal sensing device. Leveraging the fusion of respiratory timings derived from the IP signal to index breathing cycles and phases, together with simultaneously obtained LSs, we found that phase-contextualized respiratory markers and acoustic signatures exhibited changes that correlated with the transition of patients from an acute asthma attack to a compensated respiratory status state that warranted hospital discharge. In particular, the respiratory timings showed that the breathing pattern trended toward normalcy, while the phase-contextualized acoustic signatures exhibited a change toward normal LSs by T2. We further show the value of fusing these modalities for enhancing the physiological interpretation of LS spectrogram images and for enabling a simple, interpretable proof-of-concept machine learning model for risk stratification of asthma. Hence, these results highlight the feasibility of combining wearable-based respiratory acoustic features derived from LS and respiratory pattern features derived from IP for quantifying and tracking the physiological manifestations of asthma in children.

### IP and LS Features Can Quantify and Track the Resolution of Acute Asthma Signs

As the participants transitioned to a compensated respiratory status state—evidenced by the improvement in their signs enabling discharge from the PICU (Table 2)—we found that their breathing pattern changed substantially. In particular, RR decreased, and the duration of both breathing phases increased significantly. The significant reduction in RR indicates a progression toward reversing the airflow obstruction as the children were able to breathe easier. Although Te lengthening has been previously associated with airway narrowing [37], only 3 of the 13 participants showed longer Te at T1, with 2 of them also experiencing lower RR compared to T2. Furthermore, compared to T1 of the matched participants, participants in the control group exhibited significantly lower RR, longer Te, and higher Te:Ti. Hence, our phase and phase-ratio timing results seem to be largely influenced by RR. Previous studies have found strong correlations between RR and tidal breathing timings [44].

Similarly, the transition to a compensated respiratory state correlated with the LS-derived SI markers. Specifically, we found that as the participants transitioned to a more compensated state, SI100-300 decreased, while other higher-frequency subbands increased. This shift in spectral energy from low to high frequencies indicates an overall transition to a flatter and more spread-out spectrum, as we have observed on healthy participants in prior validation studies [26,27]. Notably, we recently studied forced-expiratory (FE) breathing maneuvers on healthy participants, some of which generated expiratory wheezes and found an increase in the mean roll-off frequency f95 (ie, the frequency containing 95% of the spectral energy) from 565 (SD 180) Hz in FE breaths to 929 (SD 130) Hz in normal deep breaths [27]. In addition, we observed the greatest change in the inspiratory features, particularly in the 2 uppermost frequency bands, which suggests a progressive increase in the inspiratory intensity for healthier respiratory states and supports the interpretation of a transition toward normal LS whose strongly inspiratory nature is well-known [42].

Compared to prior work, the finding that changes in spectral power between the groups did not alter the relative distribution of power within the spectral bands (ie, SI100-300>SI300-500>SI800-1000 for all groups) is in agreement with reports using this subband decomposition and normalized SIs, where the SIs were found to decrease as frequency increases regardless of the asthma severity level [39]. The SI trends from T1 found herein, however, disagree with those found by Nabi et al [39] if wheezing is assumed to be present at T1, decreased or resolved by T2, and absent in control. Comparing mild and severe asthma groups, Nabi et al [39] found that SI100-300 was reduced while the rest of the bands were increased with severity level. This discrepancy may be explained by important differences in study design and measurement conditions. First, the work by Nabi et al [39] only included high-quality segments with wheezes, while the production of wheezes, or lack thereof, was not part of the inclusion criteria in this work, nor was it used to define sign severity or disease progression. Second, prior work has used commercially available digital stethoscopes with noise-cancelling capabilities and transducers designed to aggressively filter out high frequencies [45], as illustrated in Figure 3, with the sharp drop in spectral energy after 500 Hz for the sounds measured with the Eko CORE.

In the context of asthma, the generation of wheezes is related to changes in airway thickness [46,47] and the narrowing of the air caliber leading to fluttering of the airway walls and fluids [47]. This leads to sounds exhibiting greater acoustic energy at frequencies higher than 300 Hz compared to normal LS [40,48-50]. A transition from wheezes to normal sounds would then be expected to result in SI100-300 increasing and the other SIs decreasing when using a digital stethoscope since the normal LS spectra will be inherently concentrated below 300 Hz by the sensor itself. In our case, however, the passive nature of the acoustic measurements leads to normal LS becoming flatter, compared to wheezes, rather than more concentrated at lower frequencies, with the ultimate result of decreasing SI100-300 and increasing the other subband’s SIs, as the spectral energy becomes spread out between the other bands. Supporting this argument are the opposing trends in f75 and f95 (ie, the frequencies containing 75% and 95% of the total spectral energy, respectively) observed between the Eko CORE and WIRS when comparing normal deep breaths and FE breaths, wherein normal deep breaths resulted in both f75 and f95 increasing for WIRS but decreasing for Eko CORE [27].

Finally, the matched cohort analysis further confirmed the above observations, highlighting that the direction of change in all IP and LS markers across T1 and T2 was toward a compensated respiratory status (control). This suggests the progressive resolution of the acute asthma signs, wherein participants at T2 are physiologically closer to healthy individuals (ie, control group). However, the differences in RR, Te:Ti, and Ti/IBI between T2 and control indicate that such a transition was not complete before PICU discharge. This may be explained by participants transitioning from the PICU to a regular hospital room before going home for further monitoring until recovery. Hence, their respiratory status at T2 may not have reached normalcy. In all, these results suggest that parameters derived from wearable-based IP and LS may be effectively used to quantify and track respiratory status from peak to resolution of an acute asthma attack.

### Wearable-Based Multimodal Respiratory Features May Enable Robust Machine Learning Models for Risk Stratification of Asthma

In the proof-of-concept PCA, we found that PC1 captured close to 61% of the total variance in the data and was significantly higher for control compared to T1 and T2 for the matched cohort. The ranking further reveals that the transition toward a compensated respiratory status was most strongly influenced by increased intensity in the 500-800 Hz subband, decreased intensity in the 100-300 Hz subband, increased intensity in the 800-1000 Hz subband, and finally, increased intensity in the 300-500 Hz subband. Expiratory features showed higher ranks compared to their inspiratory counterparts, but the differences were marginal. This finding is consistent with those reported in Nabi et al [39], wherein inspiratory and expiratory phases were found to be equally informative for discriminating severity levels. Indeed, airway obstruction in asthma may lead to both inspiratory and expiratory LS abnormalities [40]. In the context of children with asthma, inspiratory and expiratory wheezes may be equally informative for characterizing asthma severity levels [51]. Nevertheless, the ability to segment wearable-based LS recordings into phases was enabled by the simultaneously measured IP signal, which motivates further investigation into their utility for monitoring children with asthma. Overall, the feature ranking revealed that the LS-derived phase-specific SI features were more important than the IP-derived features. This highlights the importance of quantifying and tracking respiratory acoustic features in combination with respiratory pattern features for monitoring asthma in children.

### The Combination of IP and Multichannel Breath Sounds Holds Promise for Continuous Monitoring of Asthma Signs

Although the IP-derived features ranked lower in importance in our PCA, these results nevertheless highlight the potential of combining both sensing modalities for continuous monitoring of asthma. IP enables the respiratory contextualization of the sounds while also enabling the calculation of well-known tidal breathing parameters, many of which have shown promise for monitoring children with asthma [19-21,25,52]. Furthermore, the combination may not only enhance the discriminatory power of ML models but also the physiological interpretation of the results [27]. Examples of this are our findings of polyphonic expiratory wheezes (Figure 3) and the abrupt transitions in SI in 2 of the sickest participants of the cohort (Multimedia Appendix 1). In the former, the finding indicates that the patient was likely obstructed at T1 and that the reversal of the obstruction resulted in normal LSs at T2 [40,53]. Indeed, the clinical records indicate that this 3-year-old patient transitioned from high-flow nasal cannula at T1 to no ventilatory support at T2 and was discharged home at the same time as PICU discharge. Similarly, the albuterol dosing interval increased from 3.4 to 4.3 hours. Hence, the resolution of expiratory wheezes observed in this patient is consistent with the transition to a normal, compensated respiratory state. In the latter, the expiratory sounds found therein are consistent with snoring or rhonchi that likely resolved after awakening and repositioning. Notably, expiratory snoring and rhonchi may be indicative of airflow obstruction [40,53,54]. In these cases, tidal breathing parameters derived from the IP signal together with phase-specific SI features provide a more holistic and interpretable picture of the physiologic manifestations of asthma in children.

### Limitations and Future Work

This study has several limitations. First, the sample size is small and may not capture the well-known disease heterogeneity and severity in asthma [3,6] and limits the assessment of correlations between physiological markers and clinical variables. Second, high intersubject variability in the respiratory markers’ response throughout hospitalization was expected in this dataset because the measurement period was different between patients, as well as their relative recovery before PICU discharge. Third, the higher variability in RRs observed in this sample required subject-specific processing parameters to ensure signal quality. This may have introduced a minor bias in the final feature matrix. Fourth, due to the inability to use a regular patient room in the PICU for healthy controls, their measurement took place in a different setting. Although measurements were equally uncontrolled, these participants were naturally freer to move and talk. Furthermore, despite our efforts to maintain the same sensor positions throughout the recordings and across participants, changes in measurement conditions from recording to recording may have resulted in sensing location variability. Likewise, measurements likely took place in slightly different postures due to variations in head-of-bed angles and participants leaning forward for auscultation on the posterior chest. Ultimately, however, this study aimed to evaluate the feasibility of recording multimodal physiological signals with a wearable with the least amount of interference to the regular hospital workflows. Hence, the measured signals captured the natural variability that occurs in realistic continuous monitoring scenarios.

We only analyzed 1 of the 4 locations measured by WIRS. Including additional channels in the analysis will provide regional information, which may improve the results further because LS intensity is known to be a function of auscultation location [40,42]. The LS signal quality evaluation consisted of removing breaths with outlying intensities after rejecting breaths based on the LS signal quality. This may have resulted in the inclusion of low acoustic-quality breaths in the analysis. Yet, there are currently no validated methods for LS signal quality assessment that can be broadly applied to any type of sounds within the vast dictionary of LS described in the literature. Moreover, notable efforts along this direction have developed features to characterize noise based on recordings measured with sensors that integrate active noise-cancellation strategies, which may be different from those obtained with custom wearable systems such as WIRS, as previously discussed. These observations underscore the need for novel signal quality assessment algorithms that not only incorporate LS data measured with wearable systems but also other wearable-based sensing modalities correlated to the generation of the sounds (ie, IP, IP-derived flow, and chest motion) [26,27,40,42,55]. The successful application of such a method may enable a robust and continuous monitoring of breath sounds at home and in otherwise uncontrolled settings.

Future work will evaluate ML models trained on more complex feature vectors that combine LS and IP data to elucidate their ability to predict sign severity in children hospitalized with asthma. Other auxiliary information obtained from multiple sensors (eg, kinematics and posture) will be evaluated to contextualize the recordings and increase robustness. Chest kinematics via an inertial measurement unit were measured by WIRS and may thus be studied in the future, given the promise shown in recent work to contextualize multimodal recordings [55,56]. Longer tidal-breathing recordings may be studied to enable the incorporation of respiratory pattern variability metrics [15]. Tidal flow variability metrics derived from tidal flow-volume loops may also be studied to assess lung function variability in children with asthma [19,20,24,52]. Furthermore, correlations to clinical outcomes will be studied on larger and more diverse datasets to elucidate the value of wearable technologies such as the one used in this work for supporting clinical decisions.

### Conclusions

We evaluated the feasibility of monitoring children from peak to resolution of acute severe asthma signs using wearable-based BioZ and multichannel breath sounds. We found that respiratory timings derived from the IP signal and breathing phase–contextualized SIs derived from the LS signal were significantly altered from peak to resolution of asthma signs, and the changes trended toward the distributions observed in healthy participants. Their combination further enhanced the separation of groups in a low-dimensional subspace. These results highlight the potential of wearable multimodal sensing systems such as WIRS for monitoring the physiologic manifestations of acute asthma in children.

## Appendix

Multimedia Appendix 1 Exemplary full-length multimodal recording.

## Abbreviations

BioZ: electrical bioimpedance
CHOA: Children’s Healthcare of Atlanta
FE: forced-expiratory
FIR: finite impulse response
IBI: interbreath interval
IP: impedance pneumography
LS: lung sound
MAD: median absolute deviation
PC: principal component
PCA: principal component analysis
PICU: pediatric intensive care unit
PSD: power spectral density
RR: respiratory rate
SI: spectral integrated intensity
Te: expiration time
Te: Ti: expiration-to-inspiration time ratio
Ti: inspiration time
WIRS: wearable impedance pneumography and respiratory sound system

## Data Availability

The datasets generated or analyzed during this study are available from the corresponding author on reasonable request.
