# Supplementary material for: Monitoring Respiratory Health in Children With Acute Asthma Using Wearable Electrical Bioimpedance and Breath Sounds: Observational Case-Control Study
Source: JMIR Mhealth Uhealth. 2026 Mar 5;14:e72979. doi: 10.2196/72979 (PMC13003210; doi:10.2196/72979)
Supplement: Multimedia Appendix 1 [file mhealth_v14i1e72979_app1.docx]

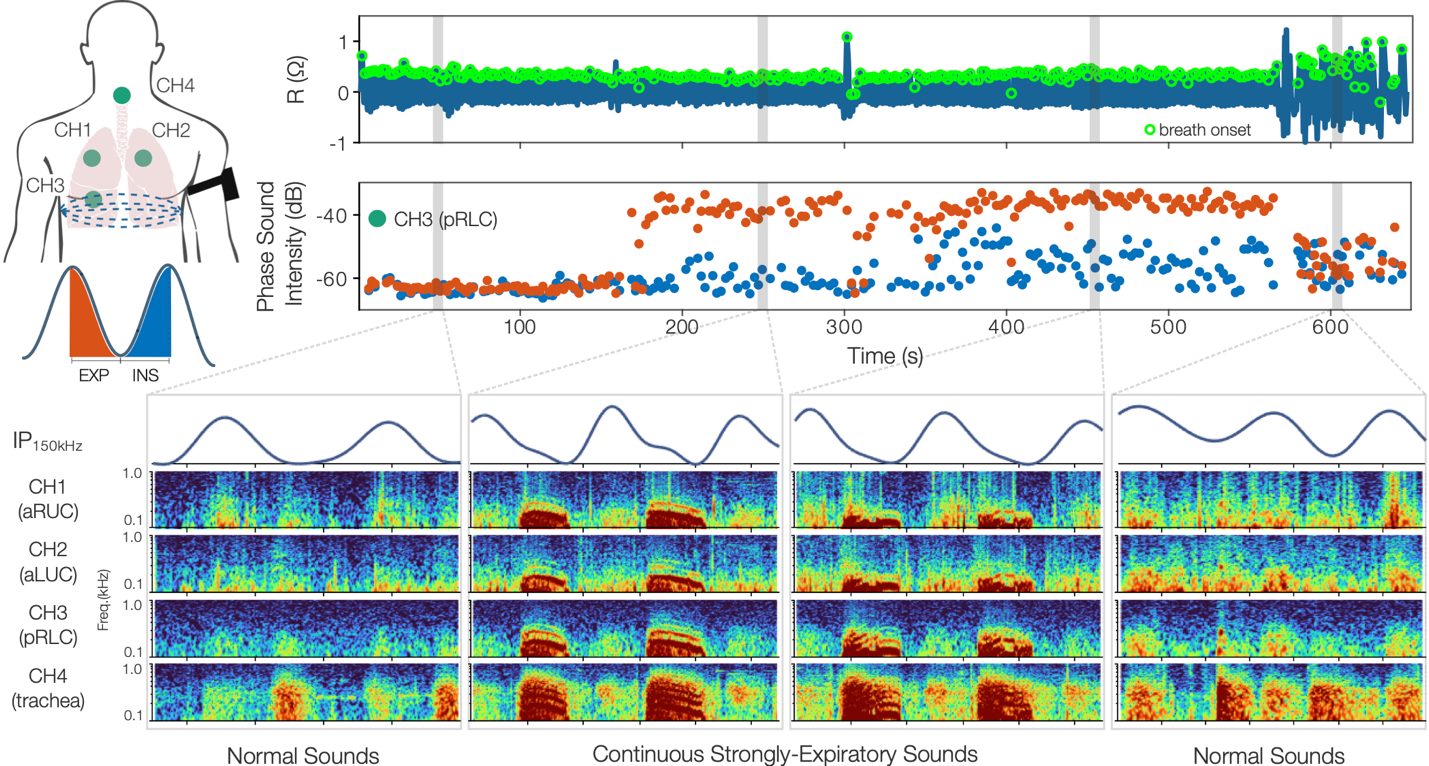


From top to bottom: Impedance Pneumography (IP) signal with annotated breath onsets, computed inspiration (INS) and expiration (EXP) phase sound intensities for CH3 and selected simultaneous IP and multi-channel breath sounds segments highlighting the differences in relative phase intensities during the recording. aRUC: anterior Right Upper Chest, aLUC: anterior Left Upper Chest, pRLC:posterior Right Lower Chest.
